# *All of Us* and the Promise of Precision Medicine: Achieving Equitable Access for Federally Qualified Health Center Patients

**DOI:** 10.3390/jpm13040615

**Published:** 2023-03-31

**Authors:** Carolyn P. Neuhaus, Danielle M. Pacia, Johanna T. Crane, Karen J. Maschke, Nancy Berlinger

**Affiliations:** 1 The Hastings Center, Garrison, NY 10524-4125, USA; 2 Alden March Bioethics Institute, Albany Medical College, Albany, NY 12208-3478, USA

**Keywords:** precision medicine, health equity, community health, FQHCs, *All of Us*

## Abstract

The United States National Institutes of Health’s (NIH) *All of Us* (AoU) initiative recruits participants from diverse backgrounds to improve the makeup of biobanks, considering nearly all biospecimens used in research come from people of European ancestry. Participants who join AoU consent to provide samples of blood, urine, and/or saliva and to submit their electronic health record to the program. In addition to diversifying precision medicine research studies, AoU will return genetic results back to many participants, which may require further follow-up care (i.e., more frequent cancer screening or mastectomy after a BRCA result). To help achieve its goals, AoU has partnered with Federally Qualified Health Centers (FQHCs), which is a type of community health center whose patient base is comprised largely of people who are uninsured, underinsured, or on Medicaid. Our NIH-funded study convened FQHC providers involved in AoU to better understand precision medicine in community health settings. Drawing from our findings, we present barriers community health patients and their providers face when accessing diagnostics and specialty care after genetic results necessitate medical follow-up care. We also propose several policy and financial recommendations to help overcome the challenges discussed, stemming from a commitment to equitable access to precision medicine advances.

## 1. Precision Medicine in Community Health Centers

Investment in precision medicine research aims to deliver targeted treatments, highly accurate diagnostics, and enhanced capacity to prevent morbidity and mortality, thus improving health outcomes. However, ensuring that this medical advancement is accessible to populations with already present barriers to health care will be challenging, particularly for patients from community health centers. Community health centers are non-profit, community-based organizations that deliver primary care to individuals and families with limited access to health care services because of geographic challenges, insurance status, or limited financial means. To help ensure that patients from community health centers have equitable access to precision medicine advances and to reduce health disparities produced by socio-economic inequalities, policy changes and more financial support for community health centers are needed.

From this perspective, we describe a national effort in the United States to recruit community health center patients to precision medicine research, focusing on the challenges these patients, their providers (e.g., physicians, nurse practitioners, and physician assistants), and community health workers face in accessing diagnostics and specialty care recommended by precision medicine when research findings indicate a need for medical follow-up. We present policy and financing recommendations to reduce or overcome these challenges and avoid foreseeable burdens and harms grounded in a commitment to deliver precision medicine equitably.

## 2. Advancing Precision Medicine Research through Community Health 

In the last decade, the United States has invested heavily in precision medicine—the clinical practice of using individual patients’ medical history, demographic factors, and diagnostic test findings, notably genetic information—to identify treatments that are likely to be effective for individual patients based in part on genetic factors [1]. A hallmark of the federal government’s investment in precision medicine is the National Institutes of Health (NIH) *All of Us* (AoU) Research Program. In 2016, Congress allocated $1.5 billion for this program over 10 years, subject to an annual appropriations process [2].

AoU is creating a cohort of one million or more residents of the United States who enroll as research participants. AoU participants answer surveys on different topics and are asked, but not required, to consent to sharing their electronic health record (EHR) with the program and to provide samples of blood, urine, and/or saliva for lab and DNA tests [3]. Once the EHR is shared with AoU researchers, key data are extracted such as medications prescribed and past medical procedures [4]. The EHR information is then standardized and stored in accordance with AoU standards in a central database [5]. Survey data and DNA analyses are similarly standardized and stored in the central database. Researchers, including citizen scientists, apply to AoU to access the database. Some AoU data are publicly available in aggregated form to help shield the identity of participants [6]. In addition to serving precision medicine research goals, AoU returns personal genetic information to participants who opt to receive it. Nonmedical information such as ancestry will be returned as well as data on traits that will not have significant medical implications (i.e., information on cilantro preferences) [7]. 

Importantly, participants will have the option to be returned medically actionable genetic and pharmacogenetic findings, necessitating follow-up care and guidance. The program’s Hereditary Disease Risk report includes germline genes and variants that are associated with serious, medically actionable health conditions such as increased risk of specific cancers, heart conditions, blood disorders, and more. AoU follows recommendations from the American College of Medical Genetics and Genomics about which variants to return to research participants [8]. Participants can also opt to receive pharmacogenomic information that include seven genes that are known to affect how the body processes certain medicines [9]. Returning medically relevant genetic information aligns with the prevailing bioethical position that research participants should have the option to be informed of genetic findings [10]. As of December 2022, AoU had returned nonmedical genetic information to more than 175,000 participants. It began returning medical information in late 2022 and returns information on a rolling basis [10]. How long participants must wait between providing samples and being returning nonmedical or medical genetic information varies. 

Federal investment in AoU was justified in part by the recognition that nearly all biospecimens used in genetics research come from people of European ancestry. This lack of diversity in genetic data from research participants is a barrier to understanding conditions that manifest disproportionately in people who are not of European ancestry and to reducing health disparities in those populations [11]. To response to these knowledge deficits, AoU aims to recruit 75% of participants from groups that have been historically underrepresented in genetics research, including racial and ethnic minorities [12]. As of December 2022, about 80% of *All of Us* participants represent communities that have been historically underrepresented in medical research, and nearly 50% of *All of Us* participants identify with a racial or ethnic minority group [9].

To achieve its ambitious recruitment targets, AoU has partnered with federally qualified health centers (FQHCs), which are nonprofit community health centers whose core mission is to deliver primary care to medically underserved geographic regions and patients [13]. The nation’s roughly 1400 FQHC systems are financed in part by the Health Center Program of the federal Health Resources and Services Administration (HRSA) [14]. FQHCs also receive payments for services from insurers and from patients on a sliding scale fee schedule, and some fundraise privately. FQHCs and similar HRSA-funded community health centers serve over 30 million people in more than 14,000 clinics in rural and urban communities across the country [15]. Most patients are uninsured or publicly insured; 21% of health center patients have private insurance. Meanwhile, 90% of community health center patients are considered to be low-income and over half are racial or ethnic minorities [16,17]. 

Partnering with AoU and other precision medicine research projects may serve FQHCs’ institutional goals, such as expanding patient access to genetic information about their health or increasing capacity to conduct research. However, what happens when AoU research participants from FQHCs receive medically actionable research results from the AoU program? How will they be able to access recommended follow-up diagnostic care related to genetic findings, given that these follow-up services are likely beyond the scope of a primary care clinic?

In our NIH-funded research project, we convened FQHC providers from AoU recruitment sites to understand the obstacles that participants from FQHCs may face when attempting to obtain follow-up services after receiving medically actionable genetic research results. We also interviewed key staff from one participating FQHC [18]. Our project was jointly funded by the National Human Genome Research Institute and the NIH’s Office of the Director, which also funds AoU. However, our research project is separate from AoU and sits within a body of qualitative research with community health centers to understand this context and its unique ethical issues. We describe in this paper the ethical and practical issues our project identified and offer recommendations for policy changes to reduce barriers to medical follow-up for community health center patients who receive medically actionable genetic results. If enacted, these changes would advance equitable access to precision medicine among publicly insured, low-income, and medically underserved people in the US.

## 3. Anxieties about Returning Genetic Research Results

Among the benefits of knowing medically relevant genetic information is that this knowledge enables people to take action before the onset of a disease or condition and permits providers to tailor medical care or interventions to a genetic finding (e.g., switching medications based on a pharmacogenetic finding, more frequent cancer screening through mammogram or colonoscopy, or regular imaging through X-ray, CT scan, or MRI). In some cases, genetic findings may support more invasive, surgical interventions, such as mastectomy for a BRCA finding. However, people who face barriers to routine health care access will also face barriers to acting on genetic knowledge. 

AoU includes some provisions to support the ability of participants to understand genetic findings, including medically actionable findings. All AoU participants are provided access to genetic counselors via telehealth, and consults are available in both English and Spanish [19]. Participants who receive a medically actionable genetic and/or pharmacogenetic finding are provided free access to clinical validation of their research results [20]. Official clinical validation is important because AoU genetic results do not meet standards associated with laboratory testing performed for the purpose of prevention or treatment of disease, per federal law [21]. 

However, even with a clinically validated result and access to genetic counselors, it is foreseeable that both the patient–participant and their provider at a community health center will have difficulty securing follow-up diagnostics and care once a medically actionable genetic or pharmacogenetic research finding is returned. As noted, the specialty services needed for clinical response to medically actionable results are usually beyond the scope of the primary care services provided by FQHCs, which include family medicine, internal medicine, prenatal care, pediatric care, preventative health, and the management of chronic conditions such as asthma, diabetes, and hypertension [13]. This means that, for an FQHC patient–participant acting on genetic knowledge may require access to services that are entirely unavailable at FQHCs.

Through our study, we learned that many FQHC leaders and frontline providers are uncertain and concerned about their institutional capacity to ensure access to recommended follow-up clinical services to their AoU participant-patients who need them. In particular, they expressed concern about how to counsel and advise uninsured and underinsured patients about recommended follow-up testing and care, given these patients’ inability to pay for these services—or, in some cases, the known reluctance of an academic medical center to accept referrals of FQHC patients who are uninsured, underinsured, or on Medicaid.

Some FQHC providers expressed sadness about not knowing what was going to happen when their patients started receiving medically actionable genetic research findings from the AoU program. We also heard that because FQHC patients have high levels of trust in their providers, FQHC providers and other staff may experience distress about a perceived breach in their trustworthiness. The concerns we documented were echoed in a separate study with providers at an FQHC in Phoenix, Arizona whose patients were recruited for precision medicine research. Providers there similarly expressed distress knowing that many underinsured patients would not be able to access follow-up care because of financial barriers [22].

Community health providers routinely struggle with the problem of how to secure access to specialty clinical services for their uninsured and underinsured patients. These providers are experienced at advocating for individual patients who need diagnostics or interventions available at local hospitals or medical practices, for example. Two things are unique about the problem of access posed by medically actionable genetic knowledge. One has been noted: that the needed services tend to be concentrated outside FQHCs in clinics and medical centers that may or may not be willing to accept FQHC referrals, or they may be located at considerable distance from the area served by an FQHC. The other unique feature of this access problem is the unclear relationship between federally funded research and federally funded care: how, then, should the immense research effort of the AoU program to build a biobank that includes diverse, medically underserved populations also respond to the problem of unequal access to follow-up care among these same communities? What (if anything) is owed to medically underserved patients who contribute their health data to precision medicine research? The success of AoU and other federally funded precision medicine research initiatives will likely increase community health providers’ engagement with genetics, as precision medicine becomes a more entrenched public health practice. AoU is testing whether the promise of precision medicine will, indeed, reach “all of us,” including those who rely on community health centers for medical care.

## 4. Obstacles to Follow-Up Medical Services

Here, we expand on some of the problems mentioned in the previous section and focus on FQHCs’ challenges in responding to those problems. Obstacles to follow-up medical services are summarized in Table 1. There is a wide variation in community health centers’ partnerships with regional hospitals or academic medical centers. Some have well-established referral patterns; others lack them completely [23]. Because of precision medicine’s relative novelty, there also is a lack of familiarity with how to integrate pharmacogenetic and genetic findings into primary care. For example, many FQHCs currently lack the ability to integrate such findings into EHRs [24]. Another study found that primary care providers “consistently report feeling unprepared to use pharmacogenetic data and cite a lack of point-of-care resources for applying test results in practice” [25].

There are also potential challenges concerning the deployment of FQHC staff to coordinate a new set of specialty services on behalf of patients with medically actionable findings. FQHCs have seen a high rate of workforce attrition since the start of the COVID-19 pandemic, which is in part because hospitals and hospital systems offer higher salaries [26]. Chronically understaffed community health centers that already provide care coordination to support primary care access do not have the means to hire more staff to coordinate care for the relatively small number of patients who have medically actionable genetic findings. These conditions mean that existing staff will be expected to do more. Lack of ready financing for activities that exceed the capacity of existing staff was also a challenge during the pandemic; for example, during the initial roll-out of the COVID-19 vaccine, FQHC staff volunteered without pay on weekends for pop-up vaccine clinics because of the absence of appropriate billing codes [27,28]. This lack of financing persisted until the allocation of federal funds dedicated to FQHC vaccination programs and a billing code specifically for vaccination was created [29].

Digital inequity is a significant barrier to follow-up on genetic findings and to full participation in AoU. We heard through our project that AoU’s online system for returning genetic results was inaccessible to patients who lacked computers or smartphones and was difficult for patients with low literacy or limited technological knowledge. FQHC staff revealed in qualitative interviews that many participants in AoU are unaware of how to electronically access information about the program or their own data and require one-on-one in-person assistance to complete web-based tasks such as surveys.

Medicaid is the main payer among community health patients who are insured [30]. Medicaid-funded services are reimbursed at a lower rate compared to private insurance programs and Medicare [31]. Medicaid is often considered an administrative burden by physicians [32]. These factors create barriers to follow-up care for Medicaid-insured FQHC patients. Because nearly all FQHC patients come from households whose income is below the federal poverty line, access to genetic services may, as noted, be restricted or completely blocked by a provider’s willingness or unwillingness to accept patients who are uninsured, underinsured, or on Medicaid. Follow-up for patients who are undocumented immigrants legally excluded from federally funded Medicaid, Medicare, and other benefits is a perennial challenge for FQHCs. The scope of specialist services differs state by state, depending on the particulars of state-funded Medicaid programs [33].

FQHCs are sited to be accessible to people who live in medically underserved areas. A patient who lives close to an FQHC or who can be served via a mobile clinic will face barriers to medical care that requires travel. Caregiving responsibilities, disability, lack of employee sick days, and lack of means to pay for transportation pose further barriers to traveling for tests and treatment that even the most skilled FQHC patient navigator cannot easily overcome. We heard providers mention that some FQHC patients need to drive over five hours to access follow-up care, in some cases crossing into a neighboring state where providers accept Medicaid or host a periodic free clinic.

Uninsured and underinsured patients face other barriers, including whether a nonprofit hospital system’s “community benefit” requirement of its tax-exempt status includes charity care, i.e., care provided at reduced or no cost. Some hospitals fulfill their community benefit requirement in part by providing specialist care and diagnostic and imaging services to uninsured and underinsured patients [34]. Others do not and may engage in aggressive billing tactics, including suing to garnish wages of low-income patients over unpaid bills [35]. The actual access of FQHC patients who need medical follow-up is therefore highly variable and contingent on the willingness of these nonprofit organizations to recognize and respond to the health care needs of people who lack the means to pay for the care they need.

## 5. Toward Sustainable Solutions

It currently falls on community health center patients in collaboration with their primary care providers to attempt to navigate these considerable barriers to medically appropriate follow-up when genetic research results are returned through AoU. This ad hoc approach is inequitable and unsustainable in the context of precision medicine, which usually requires reliable access to an academic medical center or other health system with the necessary diagnostics and specialties. Investment in diversifying precision medicine research through the enrollment of FQHC patients without ensuring that clinical precision medicine services are accessible to these same patients is ethically wrong.

Our study identified several pathways for facilitating access to recommended medical follow-up for AoU participants from FQHCs following the return of actionable genetic research results. These pathways aim to support access to follow-up by improving capacity within FQHCs and by incentivizing other health care providers to collaborate with them. More funding on the federal level, whether through HRSA or other agencies, is needed to make these pathways work. These pathways are summarized in Table 2.

Although our focus here is on improving access to follow-up among AoU research participants from FQHCs, enacting these suggestions may improve patients’ access to diagnostic and specialty services across the board, whether someone is returned a medically relevant genetic result through direct-to-consumer genetic testing, through research, or through clinical care.

### 5.1. Building Precision Medicine Capacity within FQHCs

To help ensure there are FQHC personnel adept at communicating genetic results to patients, FQHCs should be provided support to hire or contract genetic counselors. If hiring genetic counselors or genetic medicine specialists is not achievable or preferable, FQHCs should receive funding to provide genetic educational programs and/or consultancy services for primary care providers to supplement their knowledge related to genetic findings. This could be achieved by expanding existing HRSA programs that support the integration of genetics and other specialties into primary care [36,37]. 

FQHCs should be supported to develop workflows upon receipt of genetic and pharmacogenetic results, e.g., standard operating procedures and EHR integration of results. In a study led by the Mayo Clinic with an FQHC partner, the FQHC was able to successfully integrate genetic findings into the patient participants’ EHRs after clinically validating the results [38]. This effort serves as a potential model for how FQHCs and other community health centers may be able to integrate genetic findings into EHRs. Expanding funding and awareness of HRSA’s Regional Genetics Networks presents one way to achieve these goals [36].

Patient navigators at FQHCs are already adept at securing available follow-up care for patients. Funding should be allocated for FQHCs to hire more patient navigators and community health workers who can coordinate patients’ referrals to recommended care after genetic results are received. For example, these additional navigators can help direct patients to ensure diagnostic results are clinically certified and locate diagnostic or specialty services that might be available to the patient, depending on insurance status. Expanding the list of billable providers on integrated care teams presents one way to incorporate this suggestion into current financing for the community health workforce.

### 5.2. Improving Access to Diagnostic and Specialty Services beyond FQHCs

Improvements in access to existing telemedicine services can help facilitate connecting AoU participants to specialty services and clinicians. Through telehealth, genetic counselors can help guide participants who receive genetic results, advising them on the next steps such as attaining a clinically certified lab result. While AoU is providing genetic counselors and clinically certified lab results for their participants, ensuring that all participants are able to utilize these services is crucial. This may involve enhanced in-person patient navigation for low-digital literacy and low-income participants.

Telemedicine can be used to alleviate geographic and financial barriers often faced by health center patients and connect them to relevant health care providers. Telemedicine interfaces should be user-friendly to a diverse range of demographics with varying levels of internet and technological abilities and resources. Insurers should provide equal reimbursement to providers regardless of visit modality, including phone calls.

Finally, federal agencies and funders should develop, pilot, and evaluate payment models for uninsured and underinsured research participants and their families to help cover in-person diagnostic and specialty services. These payment models may come in the form of universal health insurance, expansion of existing insurance structures (Medicare and Medicare), as well as care provided in concordance with nonprofit health care centers’ community benefit requirements.

Advocates of precision medicine should find ways to incorporate these pathways into their advocacy for research. Advocates and researchers should examine ways that federal agencies such as HRSA, the NIH, and the Centers for Medicare and Medicaid Services can implement them. These initiatives should heavily involve FQHC personnel to ensure that they are appropriate with FQHC infrastructure and culture. Until policies and pathways that support access to follow-up care can be implemented, the promise of precision medicine remains beyond the reach of most community health center patients. Ensuring that such access gaps are addressed, within and outside FQHCs, is crucial to ensuring precision medicine’s benefits are available to all.

## 6. Conclusions

A key benefit to those who participate in the AoU research program is being able to obtain their actionable genetic research results. Yet participants recruited from FQHCs who receive these results may have difficulty accessing follow-up care because of the reluctance of academic medical centers and other systems to extend precision medicine to FQHC patients who are uninsured, underinsured, or on Medicaid. Feasible pathways to recommended follow-up diagnostics and care should be created and sustained to help ensure equitable access to precision medicine for FQHC AoU research participants and other medically underserved populations.

## Figures and Tables

**Table 1:** Obstacles to follow up medical services upon return of genetic information.

*Service Barriers*
Lack of readiness among FQHCs to incorporate genetic findings into primary care
*Insurance Barriers*
Lack of health insurance
Barriers to obtaining health insurance, such as immigration status
“Underinsurance” due to inadequate coverage or high cost to policyholder
Reluctance among some providers or medical centers to accept non-emergency referrals of uninsured patients or patients insured by Medicaid
*Other barriers*
Lack of access to genetic research results or to follow up telemedicine services due to “digital divide”
Lack of familiarity or comfort with specialty medical providers and institutions beyond primary care setting
Lack of time or resources to travel to follow up medical appointments

**Table 2:** Towards sustainable solutions to achieve equitable precision medicine.

*Building precision medicine capacity within FQHCs*
Fund FQHC capacity to hire or contract with genetic medicine specialists or genetic counselors
Provide genetic education training programs or consultancy services to enhance the capacity of primary care providers to incorporate genetic findings into primary care
Develop workflows to integrate genetic and pharmacogenetic findings into FQHC operations, including integration into electronic health records
Fund FQHC capacity to hire additional patient navigators
*Improving access to diagnostic and specialty services beyond FQHCs*
Improve access to existing telemedicine services provided by genetic and other specialists, including equal reimbursement to providers regardless of visit modality
Develop, pilot, test, and evaluate equitable payment models to cover in-person diagnostic or specialty services for uninsured and underinsured research participation and their family members

## Data Availability

The data presented in this study are available in ref. [18].
